# A survival analysis of socio-demographic and clinical predictors among hospitalized COVID-19 patients in Southern Iran

**DOI:** 10.1186/s12879-023-08129-8

**Published:** 2023-03-22

**Authors:** Atefeh Esfandiari, Jamileh Kiani, Batool Amiri, Marzieh Mahmoodi, Fatemeh Abbasi, Erfan Javanmardi, Ahmad Yazdanpanah, Allahkarm Akhlaghi, Hedayat Salari

**Affiliations:** 1 Department of Health Policy and Management, School of Medicine, Bushehr University of Medical Sciences, Bushehr, Iran; 2 Clinical Research Development Center, The Persian Gulf Hospital, Bushehr University of Medical Sciences, Bushehr, Iran; 3 Department of Biostatistics and Epidemiology, School of Health and Nutrition, Bushehr University of Medical Sciences, Bushehr, Iran; 4 Department of Infectious Diseases, School of Medicine, Bushehr University of Medical Sciences, Bushehr, Iran; 5 Department of Ophthalmology, School of Medicine, Bushehr University of Medical Sciences, Bushehr, Iran; 6 Department of Otorhinolaryngology–Head and Neck Surgery, School of Medicine, Bushehr University of Medical Sciences, Bushehr, Iran

**Keywords:** COVID-19, Bushehr, Iran, Registry, Mortality, Risk factors, Survival

## Abstract

**Background:**

This study aimed to evaluate the socio-demographic, clinical, and laboratory risk factors in hospitalized COVID-19 patients during the first 6 months of the SARS-CoV-2 epidemic.

**Method:**

This retrospective hospital-based cross-sectional study included all laboratory-confirmed cases of the COVID-19 virus that were admitted to the Shohadaye-Khalije-Fars Hospital in Bushehr, Iran, from February 22, 2020 to September 21, 2020. The patients' records were reviewed during the hospitalization period. The global COVID-19 clinical platform, i.e., the World Health Organization Rapid Case Report Form was used as the data collection tool. We conducted the survival analysis using the Kaplan–Meier and the Stepwise Cox regression analyses.

**Results:**

The analysis included 2108 confirmed cases of COVID-19 with a mean age of 47.81 years (SD 17.78); 56.8% men, 43.2% women and 6.3% (n = 133) deaths. After adjustment, it was found that factors associated with an increased risk of death consisted of chronic kidney disease, intensive care unit admission, cancer, and hemoptysis. The 7-day survival rate was 95.8%, which decreased to 95.1%, 94.0%, and 93.8% on days 14, 21, and 28 of hospitalization, respectively.

**Discussion and conclusion:**

Older COVID-19 patients with manifestation of hemoptysis and a past medical history of chronic kidney disease and cancer, should be closely monitored to prevent disease deterioration and death, and also should be admitted to the intensive care unit.

## Background

The COVID-19 pandemic has led to a rise in global deaths. This newly emerged disease has high transmissibility [1], so much so that about 420,000 confirmed cases with this virus were reported from February 2020 to September 2020, over 24,000 of whom died in Iran [2]. However, the number of infected cases, deaths, and mortality rates related to COVID-19 vary from country to country due to low test capacities, underreporting, case-mix of infected and deceased patients, the burden of comorbidities, and the population age structure [3]. Also, SARS-CoV-2 mutations and a host genetic factor, HLA genotypes might affect the susceptibility to SARS-CoV-2 infection or the severity of COVID-19 [4].

COVID-19 has a scale of severity that ranges from asymptomatic to mild, moderate, severe, and death. Case fatality rates demonstrate that the severity of the disease is related to predictive risk factors and the quality of healthcare [3]. Clinical characteristics of the disease and predictors of mortality have been described in patients from other countries [5]. Reports show that upon admission, having clinical manifestations and the pre-existence of chronic medical conditions such as hypertension, cardiovascular diseases, diabetes, chronic respiratory diseases, obesity, and age are possible risk factors that account for high morbidity and mortality rates among COVID-19 patients [3, 6].

In this study, we aimed to assess the demographics, comorbidities, clinical signs and symptoms, and laboratory findings using a survival analysis during the first 6 months of the 2020 outbreak in a referral hospital in the south of Iran. A better understanding of the disease in different health care settings and different countries will increase the validity and reliability of previously described knowledge and the presence of any emerging patterns in COVID-19 patients.

## Methods

### Study design, study population, and data collection

The data were extracted from the retrospective COVID-19 hospital-based registry which were previously reported in detail as the study protocol in another study by the writers [7]. This study included all laboratory-confirmed cases of the COVID-19 virus, regardless of the presence of clinical signs and symptoms, who were admitted to the Shohadaye-Khalije-Fars Hospital in Bushehr located in the south of Iran from February 22, 2020, to September 21, 2020. The Hospital was considered the referral hospital for suspected COVID-19 patients who needed hospitalization and as well as all critically ill COVID-19 patients from anywhere in the province who were in need of the intensive care unit.

The data were extracted from patient records. The demographic and general information was retrieved by trained nurses and researchers. This information was then sorted into a printed checklist and later into a Microsoft Excel sheet.

The data collection tool is the global clinical platform COVID-19 named the Rapid Core Case Report Form (CRF) of the World Health Organization [8]. The Rapid Core CRF is designed to collect data obtained through examination, interview, and review of hospital records. CRF consists of 3 modules: Module 1 is completed on the first day of hospital admission; Module 2 is completed during the hospital stay. Module 3 is completed upon the patients' discharge or death.

The data have been gathered in four parts: The demographic data includes names, age, the level of education, marital status, employment status, and the place of residence. Module 1 consists of the date of admission, vital signs, comorbidity, pre-admission medication list and chronic medications, clinical signs and symptoms at admission, medication on the day of admission, intensive supportive care including ventilators and ICU on the day of admission, and laboratory results. Module 2 includes daily follow-up during the hospital stay, vital signs, daily clinical signs, laboratory results, medications received during hospitalization, and intensive care. Module 3 completed at discharge/death, entails a diagnostic/pathogen test, a report of problems at the time of hospitalization, medication on admission or discharge, intensive care, and outcome.

### Participants

**Fig. 1:**
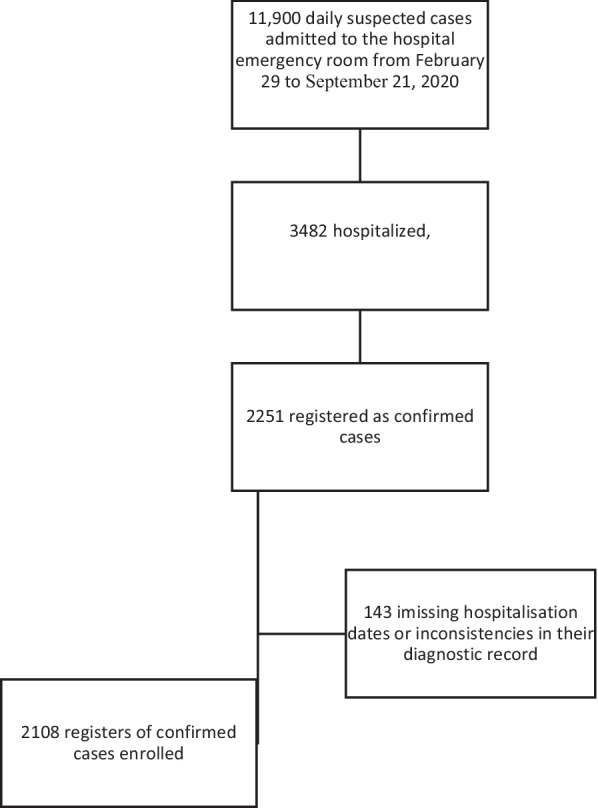
Flow diagram of the study

The study particiapnts included all COVID-19 patients admitted to the hospital during the study period (February 22, 2020, to September 21, 2020) and who were also laboratory-confirmed cases (reverse transcription-polymerase chain reaction in nasopharyngeal and deep nasal swabs). Out of 11,900 suspected cases admitted to the hospital's emergency room from February 22 to September 21, 2020, 3482 were hospitalized. Of them, 2251 were registered as confirmed cases. After excluding 143 cases who had missing hospitalization records or had inconsistencies in their diagnostic records, 2108 confirmed cases enrolled in the study. The flow diagram of the study is shown in Fig. 1.

We assured the participants of the confidentiality of their information. Once the data were received, they were closely checked for completeness and accuracy and when they were computerized, they were again checked for their consistency.

### Statistical methods

Microsoft Excel 2016 was used for data entry, and then the data were exported to SPSS version 24 for further analysis. Before analysis, the data were cleaned. Histograms and the bias-kurtosis and Kolmogorov–Smirnov tests were used to evaluate the normality. Since variables had a normal distribution, comparisons between individuals who had died versus those who had survived were made through the independent samples t-test for counting variables, and X^2^ for categorical variables. The data were analyzed based on the estimates of survival functions, using the non-parametric Kaplan–Meier method. A Cox proportional hazards regression model was used to determine factors associated with death time. A stepwise analysis was conducted by entering the variable if p-value < 0.05 and removing the variable if p-value < 0.10. The Adjusted Hazard Ratios (AHR) with 95% confidence intervals were computed, and the statistical significance was confirmed when it was significant at a 5% level (p-value < 0.05). We defined death as occurrence of in-hospital death. The exclusion criteria included patients who their medical records were incomplete.

This study was approved by the Ethical Committee of Bushehr University of Medical Sciences with the ethical approval code: IR.BPUMS.REC.1399.005.

## Results

The analysis included 2108 confirmed cases of COVID-19. The age range of the patients was 1 month to 98 years and the mean age was 47.81 ± 17.79 years. 1.37% (n = 29) were children(under the age of 13). 56.8% (n = 1197) were men, 43.2% women,and 10.9% (n = 230) were admitted to the ICU. In addition, 3.3% of the patients were the medical care staff and 0.2% were the laboratory staff. Until the end of the study period, 6.3% of the patients (n = 133) died, 92.0% (n = 1939) of the cases were discharged or recovered, 4 patients (0.2%) remained hospitalized and 32 patients (1.5%) were transferred to other locations. There were no death in children under the age of 13. Tables 1 and 2 demonstrate the clinical and laboratory characteristics of the study population at the time of admission. The median duration of hospitalization in total, for cases of death, and for cases of recovery was 4 days (IQR: 1–7), 5 days (IQR: 2–10), and 4 days (IQR: 1–6), respectively.

**Table 1:** Socio-demographic and clinical characteristics of the study population

Variable	Total patient n = 2108(%)	Dead n = 133(%)	Alive n = 1975(%)	p-value
Age	47.81 ± 17.78	59.90 ± 19.04	27.10 ± 17.40	< 0.001^a^
Gender				
Male	1197 (56.8)	82 (61.7)	1115 (56.5)	0.241^b^
Female	911 (43.2)	51 (38.3)	860 (43.5)	
Comorbidity	887 (42.1)	92 (69.3)	795 (40.2)	< 0.001^b^
Chronic heart disease	306 (14.5)	56 (42.5)	250 (12.6)	< 0.001^b^
High blood pressure	159 (7.5)	16 (12.0)	143 (89.94)	0.043^b^
Chronic lung disease	38 (1.8)	7 (5.2)	31 (1.6)	0.002^b^
Asthma	78 (3.7)	6 (4.5)	72 (3.6)	0.609^b^
Chronic kidney disease	50 (2.4)	11 (8.3)	39 (2.0)	< 0.001^b^
Diabetes	111 (5.3)	13 (9.8)	98 (5.0)	0.016^b^
Cancer	25 (1.2)	6 (4.5)	19 (1.0)	< 0.001^b^
Smoking	43 (2.0)	5 (3.9)	38 (19.2)	0.147^b^
Number of breaths per minute (≥ 24)	51 (2.4)	11 (8.9)	40 (2.0)	< 0.001^b^
Heart rate per minute (≥ 125)	114 (5.4)	6 (4.5)	108 (5.5)	0.637^b^
Systolic blood pressure (mmHg)	127.27 ± 19.67	123.35 ± 19.84	127.51 ± 19.67	0.024^a^
Diastolic blood pressure	80.40 ± 11.64	77.58 ± 11.58	80.68 ± 11.93	0.006^a^
Fever	1058 (50.2)	56 (42.1)	1002 (50.7)	0.054^b^
Cough with sputum production	365 (17.3)	22 (15.9)	343 (17.4)	0.808^b^
Cough with hemoptysis	40 (1.9)	7 (5.3)	33 (1.7)	0.011b
Sore throat	116 (5.5)	2 (1.5)	114 (5.8)	0.037^b^
Diarrhea	129 (6.1)	7 (5.3)	122 (6.2)	0.670^b^
Myalgia	163 (7.7)	7 (5.3)	156 (7.9)	0.271^b^
Headache	263 (12.5)	3 (2.3)	260 (13.2)	< 0.001^b^
Nausea and vomiting	211 (10.0)	11 (8.3)	200 (10.2)	0.490^b^
Fatigue	133 (6.3)	5 (3.8)	128 (6.5)	0.211^b^
Hospitalization in the ICU	230 (10.9)	109 (81.9)	121 (6.1)	< 0.001^b^
CRP				
≤ 10	1553 (73.7)	120 (90.2)	1433 (72.6)	< 0.001^b^
> 10	555 (26.3)	13 (9.7)	542 (27.4)	
LDH				
≤ 436	942 (44.7)	21 (15.8)	921 (46.6)	< 0.001^b^
> 436	1166 (55.3)	112 (84.2)	1054 (53.4)	
SpO2				
≤ 93	333 (15.8)	71 (53.4)	262 (13.3)	< 0.001^b^
> 93	62 (46.6)	1713 (86.7)		
WBC count (× 10^3^/L)				
< 4	239 (13.9)	8 (6.0)	285 (14.4)	< 0.001^b^
4–10	1174 (55.7)	58 (43.6)	1116 (56.5)	
> 10	641 (30.4)	100 (75.2)	541 (27.4)	
CT (Bilateral pulmonary infiltration)	1720 (81.6)	130 (97.7)	1590 (80.5)	< 0.001^b^
Number of hospitalization days	5.16 ± 7.24	7.19 ± 6.90	5.08 ± 7.36	0.002^a^

^a^Independent samples T-test

^b^X^2^ test

**Table 2:** Laboratory characteristics of the study population

Variable	Group	Mean	Std. deviation	p-value
SpO2_3	Live	95.30	6.00	0.601
	Dead	96.00	4.74	
Hemoglobin (g/L)	Live	13.31	23.21	0.922
	Dead	12.81	2.22	
WBC count (× 10^3^/L)	Live	8.84	20.43	0.603
	Dead	6.40	2.045	
Hematocrit (%)	Live	40.83	36.36	0.563
	Dead	36.33	15.06	
Platelets (× 10^3^/L)	Live	215.87	70.83	0.702
	Dead	213.02	76.11	
APTT/APTR	Live	27.76	23.07	0.413
	Dead	21.43	12.71	
PT (s)	Live	15.07	8.54	0.692
	Dead	14.09	7.27	
INR_3	Live	1.49	2.33	0.570
	Dead	1.13	0.18	
ALT/SGPT (U/L)	Live	42.98	53.60	0.965
	Dead	43.60	25.12	
Total bilirubin (µmol/L)	Live	1.73	8.05	0.626
	Dead	0.68	0.809	
AST/SGOT (U/L)	Live	40.64	40.52	0.723
	Dead	44.25	21.84	
Urea (BUN) (mmol/L)	Live	18.09	17.92	0.201
	Dead	13.06	7.88	
Creatinine (μmol/L)	Live	1.71	6.57	0.651
	Dead	1.05	0.33	
Sodium (mEq/L)	Live	135.39	53.56	0.859
	Dead	137.47	3.82	
Potassium (mEq/L)	Live	6.49	28.72	0.709
	Dead	4.03	0.43	
CRP (mg/L)	Live	48.09	18.453	0.895
	Dead	41.80	40.63	
LDH (U/L)	Live	444.08	235.28	0.112
	Dead	545.35	207.23	
Troponin (ng/mL)	Live	1.03	9.19	0.784
	Dead	0.00	0.00	
ESR (mm/h)	Live	38.16	29.29	0.113
	Dead	49.03	30.44	

**Fig. 2:**
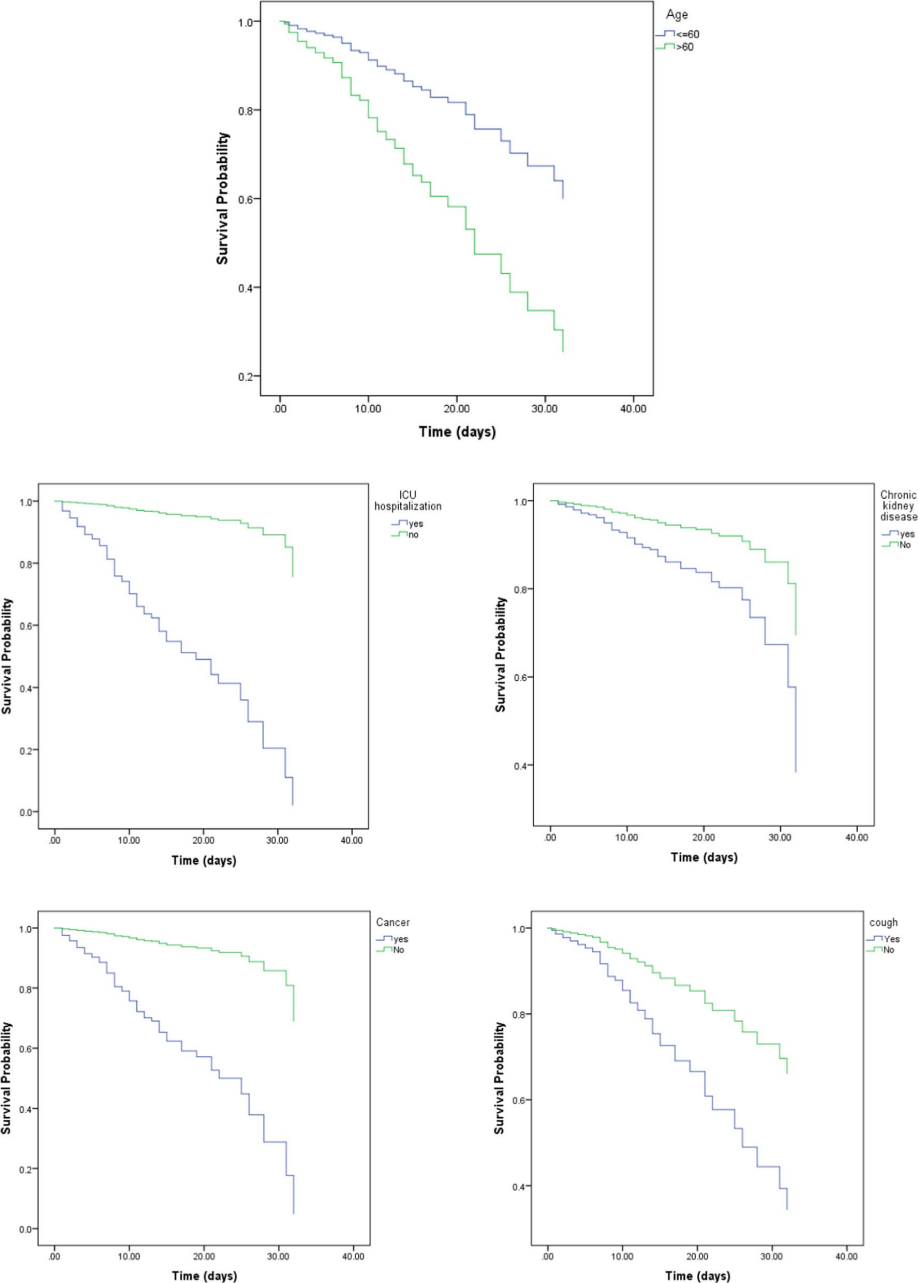
The Kaplan–Meier survival plots for the prognostic factors

The Kaplan–Meier survival plots for the prognostic factors that returned statistically significant results are presented in Fig. 2. The proportional hazards assumption is satisfied since the survival risk curves do not cross during the survey period. As can be inferred from the plot, the risk is directly proportional to the age group. Moreover, the subjects who were older than 60 years had about 25% less probability of survival after 20 days of hospitalization than those who were younger than 60 years. The subjects who were admitted to the ICU, had about 40% less probability of survival after 20 days of hospitalization than those who did not enter this unit. The subjects with cancer had an approximately 45% lower probability of survival after 20 days of hospitalization than those who did not manifest these characteristics. The patients who had hemoptysis had about 17% less probability of survival after 20 days of hospitalization than those who did not. Finally, the subjects with underlying kidney diseases were less likely to survive than those without such diseases.

In general, the 1-day survival rate was 98.4%, which decreased to 95.8%, 95.1%, 94.0%, and 93.8% on days 7, 14, 21, and 28 of hospital stay, respectively. The 1-day survival rate of patients admitted to the ICU was 91.1%, which decreased to 72.6%, 60.0%, 55.8%, and 53.7% on days 7, 14, 21, and 28 of hospital stay, respectively. With patients who died, the 1-day survival rate was 74.8%, which decreaed to 33.9%, 11.8%, 5.5%, and 1.6% on days 7, 14, 21, and 28 of hospital stay, respectively. These results showed that the survival rate of the deceased patients is lower than that of living patients. We conducted the comparison between patients in the ICU and the general wards.

A Cox proportional hazards regression model was used to determine the factors associated with the time of death. The HR from multivariable Cox proportional hazards regression models is reported in Table 3. In multivariable analyses, the following factors were associated with the risk of death: Age (P = 0.016), kidney diseases (P = 0.015), cancer (P < 0.001), hemoptysis (P < 0.001) and admission to ICU (P < 0.001).

**Table 3:** Multivariable Cox

Variable	B	SE	Wald Statistic	df	P-value	HR	95.0% CI for HR	
							Lower	Lower
age level > 60	0.55	0.23	5.83	1	0.016	1.73	1.109	2.707
kidney disease (yes)	0.76	0.31	5.97	1	0.015	2.14	1.163	3.952
Cancer (yes)	2.55	0.51	25.53	1	< 0.001	12.74	4.748	34.203
Cough with hemoptysis (yes)	1.49	0.34	18.62	1	< 0.001	4.25	2.204	8.208
ICU (yes)	2.71	0.30	83.20	1	< 0.001	15.06	8.408	26.967

Other variables were excluded from the model due to having probability values higher than 0.10 and lack of having a statistically significant effect at the level of 0.05. The risk of death was notably higher for people older than 60 years old. They had 1.73 (CI: 1.109–2.707) times a higher risk of death compared with the those younger than 60 years. The subjects with kidney diseases were 2.14 (CI: 1.163–3.952) times more likely to die compared with those without such diseases. Furthermore, those who had cancer were 12.74 (CI: 4.748–34.203) times more likely to die compared with those without this disease. Furthermore, patients with hemoptysis were 4.25 (CI: 2.204–8.208) times more likely to die compared with patients without these signs and symptoms. Finally, patients admitted to the ICU were 15.06 (CI: 8.408–26.967) times more likely to die compared with those who did not enter these wards.

$$\begin{array}{ll} {\text{h }}\left( {{\text{t }}|{\text{X}}} \right) \, = {\text{ h }}0\left( {\text{t}} \right).{\text{ exp }}\left( {{2}.{\text{72 ICU}}.{\text{HDU}}} \right) \hfill \\ {\text{h }}\left( {{\text{t }}|{\text{X}}} \right) \, = {\text{ h }}0\left( {\text{t}} \right).{\text{ exp }}\left( {0.0{3}0{\text{ Age }} + { 2}.{\text{596 ICU}}.{\text{HDU}}} \right) \hfill \\ {\text{h }}\left( {{\text{t }}|{\text{X}}} \right) \, = {\text{ h }}0\left( {\text{t}} \right).{\text{ exp }}\left( {0.0{\text{29 Age }} + { 1}.{5}0{\text{ Cancer }} + { 2}.{6}0{\text{2 ICU}}.{\text{HDU}}} \right) \hfill \\ {\text{h }}\left( {{\text{t }}|{\text{X}}} \right) \, = {\text{ h }}0\left( {\text{t}} \right).{\text{ exp }}\left( {0.0{\text{28 Age }} + { 2}.0{\text{62 Cancer }} + - {1}.00{\text{ hemoptysis }} + { 2}.{\text{69 ICU}}.{\text{HDU}}} \right) \hfill \\ {\text{h }}\left( {{\text{t }}|{\text{X}}} \right) \, = {\text{ h }}0\left( {\text{t}} \right).{\text{ exp }}\left( {0.{\text{55 Age }} + 0.{\text{76 kidney disease }} + { 2}.{\text{55 Cancer }} + { 1}.{\text{49 hemoptysis }} + { 2}.{\text{71 ICU}}.{\text{HDU}}} \right). \hfill \\ \end{array}$$

Finally, the Cox regression model was estimated as the last line according to the regression coefficients of the effective variables.

Furthermre, we assessed the relation between SpO2 and underlying diseases and age in COVID-19 patients. The results showed that although none of the underlying diseases were significantly associated with SpO2, age was significantly associated with it (P < 0.001). The average age of patients with SpO2 ≤ 93 was 53.10 ± 20.54 and the average age of patients with SpO2 > 93 was 42.35 ± 16.83.

## Disscussin

This study aimed to determine the duration of hospitalization until death as a result of the Wuhan's coronavirus disease as well as its predictors among patients admitted to the Shohadaye-Khalije-Fars Hospital. In the alpha variant COVID-19 epidemic in Bushehr, the in-hospital mortality rate among patients with COVID-19 was 6.3%. Our study findings are consistent with a systematic review which reported a range from 1 to 52% of hospital admissions; however, they are not compatible with the findings of a meta analysis which revealed that the pooled prevalence of the in-hospital mortality in patients with coronavirus disease was 15% (95% CI: 13–17) [9]. This can be due to mobilization of adequate resources to this reference hospital in the Bushehr province at the time, which prevented the hospiotal from becoming overwhelmed with patients. In addition, the presence of skilled health care providers in this educational hospital may be the other reason.

In general, the 7-day survival rate was 95.8%, which decreased to 95.1%, 94.0%, and 93.8% on days 14, 21, and 28 of hospital stay, respectively. Therefore, the first week of admission is a crucial time for the patient's prognosis and must be monitored closely for any prescribed intervention. The median values for the duration of hospitalization in total, for cases of death, and for survival cases were also very close (4 to 5 days) may reflecting the fact that the patient's status may deteriorate in earlier days of hospitalization. Regarding this disease's natural course and lack of proven treatment in the early days of the pandemic, this finding may reflect the hesitation of a patient in going to hospital as observed in a study conducted in hospitals in Brazil [10]. This can be due to unknown, stigmatized, and dread labeling of the COVID-19 pandemic, capturing cognitive and emotional aspects of people’s concern, ending with delayed hospital attention.

Factors associated with an increased risk of death were age, hemoptysis, chronic kidney disease, intensive care unit admission, and cancer. Therefore, interventions to increase the survival rate have to focus on older adults with comorbidities. Similar results have been reported in other parts of the world including China, Italy, Mexico, USA [10–12]. The weaknesses of older people may be related to having a weaker immune system and the prolonged pro-inflammatory response. They cause damage to different parts of the body that highly express angiotensin-converting enzyme genes, such as the heart and lungs [10, 11]. Contrary to expectations, this research did not find a significant difference between males and females, which is also a risk factor reported previously [1, 10, 11, 13]. Nijman et al. also did not find an increased risk of death in patients of the male sex [14]. The most likely explanation for the negative result could be psychosocial, cultural, and biological aspects.

The data obtained is broadly consistent with the major trend that comorbidity is a major contributor to death due to COVID-19 [1, 10, 11, 13–21]. Chronic kidney disease (adjusted HR 2.14; 95% CI 1.425–4.867) and cancer (adjusted HR 12.74; 95% CI 2.787–23.787) was associated with lower death rates in severe COVID-19 patients, which seems to be similar to the rest of the world [1, 10, 21]. No significant difference was found in the survival curves of the diabetes co-variable, which have been reported previously by Nijman et al. similarly [14]. Be that as it may, diabetes is also a risk factor that has been reported previously.

As with clinical characteristics, signs or symptoms as the other study variables, hemoptysis on admission was statistically significant. Similar to our finding, in other study the researcher found that hemoptysis (OR = 4) had a different distribution in two groups of severe cases and mild cases [22]. The prevalence of hemoptysis was 1.9% among the patients in our study which is close to several studies [10, 23, 24].

As expected, the major predicting factor of death was intensive care unit admission (adjusted HR 15.06; 95% CI 7.559–25.036). As ICU admission is the indicator of the high-level severity of the disease, it strongly represents an increased risk of death.

## Strengths and limitations

The main strengths of this study are inclusion of only laboratory-positive cases, the half-year of the pandemic crisis case coverage, and the advanced statistical model for analysis. However, the study was faced with some limitations, too. First, the recall bias of self-reported pre-hospitalization information is probable. Second, since missing data on some variables were excluded from the analysis, this may reduce the representativeness of the samples. Third, in the beginning of the epidemic, the diagnostic value of D-dimer and IL-6 was not proved, yet. On the other hand, due to economic conditions, and lack of facilities in the hospital, it was not checked in our center. Furthermore, it was not possible to send samples to more equipped laboratories. Fourth, the checklist used was WHO-Version 8 April 2020—revised 13 July 2020. There was no WBC diff, lymphocyte count in this checklist. So, lymphopenia was not reported in our study. Of course, WBC was considered in the checklist. At last, the absence of data on some of the COVID-19 patients is probable.

## Conclusion

This study aimed to evaluate the socio-demographic, clinical, and laboratory risk factors in hospitalized COVID-19 patients. Older COVID-19 patients with manifestation of hemoptysis and a past medical history of chronic kidney disease and cancer, should be closely monitored to prevent disease deterioration and death, and also should be admitted to the intensive care unit.

## Data Availability

The datasets used and/or analysed during the current study are available from the corresponding author on reasonable request. The data were obtained from hospital records as described in the paper.

## Abbreviations

COVID-19: Coronavirus Disease 2019
HLA: Human leukocyte antigen
ICU: Intensive Care Unit
HR: Heart rate
